# The two-year incidence of hip osteoarthritis after arthroscopic hip surgery for femoroacetabular impingement syndrome

**DOI:** 10.1186/s12891-019-2646-5

**Published:** 2019-06-01

**Authors:** Daniel I. Rhon, Tina A. Greenlee, Charles Dennis Sissel, Michael P. Reiman

**Affiliations:** 1 Physical Performance Service Line, G 3/5/7, US Army Office of the Surgeon General, Falls Church, VA USA; 2 Baylor University, Road, Bldg 2841, Suite 1301; JBSA Fort Sam Houston, Stanley, TX 3630 USA; 3 Center for the Intrepid, Brooke Army Medical Center, 3551 Roger Brooke Drive, San Antonio, TX USA; 4 Program Analysis & Evaluation, US Army Medical Command, San Antonio, TX USA; 5 Department of Orthopaedic Surgery, Duke University Medical Center, Durham, NC USA

**Keywords:** Hip pain, Osteoarthritis, Arthroscopy, Femoroacetabular impingement syndrome

## Abstract

**Background:**

One of the reported goals of hip preservation surgery is to prevent or delay the onset of osteoarthritis. This includes arthroscopic surgery to manage Femoroacetabular Impingement (FAI) Syndrome. The purpose of this study was to describe the prevalence of clinically-diagnosed hip OA within 2 years after hip arthroscopy for FAI syndrome, and 2) determine which variables predict a clinical diagnosis of OA after arthroscopy.

**Methods:**

Observational analysis of patients undergoing hip arthroscopy between 2004 and 2013, utilizing the Military Health System Data Repository. Individuals with prior cases of osteoarthritis were excluded. Presence of osteoarthritis was based on diagnostic codes rendered by a medical provider in patient medical records. Adjusted odds ratios (95% CI) for an osteoarthritis diagnosis were reported for relevant clinical and demographic variables.

**Results:**

Of 1870 participants in this young cohort (mean age 32.2 years), 21.9% (*N* = 409) had a postoperative clinical diagnosis of hip osteoarthritis within 2 years. The 3 significant predictors in the final model were older age (OR = 1.04; 95%CI = 1.02, 1.05), male sex (OR = 1.31; 95%CI = 1.04, 1.65), and having undergone an additional hip surgery (OR = 2.33; 95% CI = 1.72, 3.16). Military status and post-surgical complications were not risk factors.

**Conclusion:**

A clinical diagnosis of hip osteoarthritis was found in approximately 22% of young patients undergoing hip arthroscopy in as little as 2 years. These rates may differ when using alternate criteria to define OA, such as radiographs, and likely underestimate the prevalence. A more comprehensive approach, considering various criteria to detect OA will likely be necessary to accurately identify the true rates. Females were at lower risk, while increasing age and multiple surgeries increased the risk for an OA diagnosis. OA onset still occurs after “hip preservation” surgery in a substantial number of individuals within 2 years. This should be considered when estimating rates of disease prevention after surgery. Prospective trials with sound methodology are needed to determine accurate rates and robust predictors of osteoarthritis onset after hip preservation surgery.

## Introduction

Osteoarthritis (OA) of the knee and hip is a leading cause of disability worldwide, [1] and a primary cause of medical separation from the military [2]. Several cross-sectional and longitudinal studies suggest that femoroacetabular impingement (FAI) syndrome is a precursor to future radiographic OA of the hip [3–6]. Well-designed epidemiological studies that can adequately assess this relationship using other diagnostic criteria and in a younger adult population, are lacking [7]. Certain morphologic features of FAI Syndrome, particularly a large alpha angle, are suggested to predispose select patients to radiographic progression of hip OA [8, 9]. Surgical correction is a common treatment approach utilized to alleviate the symptoms thought to arise when repetitive bony morphological abutment leads to labrum and cartilage damage [10–12]. This surgical correction is often called a ‘hip preservation’ procedure, considered to preserve the joint by preventing or at least slowing down the progression of OA in the femoroacetabular joint [13, 14]. It is unknown if this intervention alters the onset of OA beyond morphologically-normal controls [15].

The aim of delaying the onset and progression of hip OA has in part contributed to an exponential rise in the rate of arthroscopic surgery for FAI Syndrome, [16, 17] despite the lack of long-term outcomes, and inconclusive determination regarding whether surgical intervention can influence the natural history and progression of hip OA [13, 14]. A recent systematic review reported weak evidence that surgical intervention may actually be associated with the structural progression of hip OA [18].

Determining the cost, impact on progression to degenerative joint disease, and benefit of surgery is needed to facilitate appropriate clinical decision-making [18]. A better understanding of the prevalence of OA after surgery in individuals without a pre-surgical diagnosis is necessary to improve estimates of disease onset and progression after surgery. Because radiographic evaluation of the hip may have limited reliability, [19] and due to the high rate of asymptomatic individuals with radiographic evidence of both FAI [20] and hip osteoarthritis, [21] expanding the scope of surveillance variables beyond radiographs is likely necessary to improve our understanding of this relationship. Therefore, the aims of this study were to: 1) describe the prevalence of a clinical diagnosis of hip OA within 2 years of hip arthroscopy for FAI Syndrome, and 2) determine which variables were significantly associated with a clinical diagnosis of OA after surgery.

## Methods

### Study design

The study was an observational cohort of patients seen within the U.S. Military Health System (MHS) that underwent arthroscopic hip surgery between 30 June 2004 and 1 July 2013.

### Setting

Data were derived from the MHS Data Repository (MDR), which captures and tracks all medical visits for all Department of Defense (DoD) beneficiaries. This includes retired and active service members and their families. The MDR is the centralized data repository that captures, archives, validates, integrates and distributes Defense Health Agency (DHA) corporate healthcare data worldwide. Any medical visit, in a military or civilian setting, where the DoD insurance plan is the payer (covering 100% of armed services personnel and their dependents) is captured in the MDR.

### Participants

We identified patients undergoing hip arthroscopy specifically for FAI Syndrome. Subjects under 18 or over 50 years of age were excluded, as this best represents the age range of adults that is most likely associated with FAI Syndrome (young to middle-age adults) [22]. It also represents the demographic age range of active duty service members. Because FAI Syndrome does not have its own International Classification of Diseases (ICD) diagnosis code, we identified procedures most often used to surgically treat this condition. Any subject with a recorded encounter including an arthroscopic surgical hip procedure, identified by Current Procedural Terminology (CPT) codes of 29914, 29915, 29916, or 29,862, was eligible for inclusion in the cohort. These codes identify the medical procedures rendered and are entered into individual electronic medical records. Individuals with loose body removal, diagnostic or exploratory arthroscopy codes alone (CPT codes 29860, 29861, 29863) were excluded. The codes were validated by surgeons performing a high volume of these procedures in the MHS, and these specific procedure codes have also been recommended for use in research [23]. All subjects with potential confounding diagnosis codes present prior to the surgery, which could otherwise rationalize the need for arthroscopic hip surgery, were excluded (hip avascular necrosis or infection, hip or pelvis fracture, a neoplasm or systemic arthropathy). Anyone with a prior diagnosis of OA was also excluded. Finally, all patients that were not eligible beneficiaries in the DoD health insurance and treatment plan for 12 months before and 24 months after surgery were also excluded to allow for a minimum of 2-year follow-up. Details of the extraction for the cohort have been published and are available [24].

### Reporting guidelines

The *REporting of studies Conducted using Observational Routinely collected health-Data* (RECORD) statement, [25] an extension of the Strengthening of Reporting of Observational Studies in Epidemiology (STROBE) reporting guidelines, was used to guide reporting of this study. Ethical approval for the study was granted by the Brooke Army Medical Center Institutional Review Board.

### Data sources/measurement

Person-level data for all outpatient and inpatient medical visits, both in military and civilian hospitals is captured in the MDR. The data from the MDR reflects a single-payer system, compared to the more common third-party insurance-based system. MDR internally validates data through a system where all files initially enter MDR raw, and missing data elements are continuously run across multiple other data sources in order to impute missing variables. There are over 260 sources worldwide that feed into MDR. Raw data is validated against other databases internally within MDR, before being finalized, after 90 days from initial ingestion into MDR [24]. The MDR database is proprietary to the US DHA and requires a signed Data Sharing Agreement for access to any data. The methodology for data capture and rationale for use of variables has been explained previously in detail [24].

#### Study variables

##### Descriptive variables

Patient characteristics included age, sex, military service status (military or civilian), socioeconomic status (categorized at the family unit level as military rank is associated with level of education and salary), location of surgery (military or civilian network hospital), and healthcare variables (utilization of rehabilitation and opioid pain medication). Postoperative opioid use in this cohort was defined as having 3+ unique prescriptions in the 1 year following surgery, not including the immediate perioperative dose. In recognition that certain healthcare variables could also potentially influence outcomes, we also identified relevant surgery variables (procedure type), comorbidities present after surgery (insomnia, systemic arthropathy, substance abuse, mental health), complications (infection, avascular necrosis, fracture, heterotopic ossification), and additional hip surgeries. We compared the incidence of these events between both groups. Specific codes used to identify variables and relevance in individuals with musculoskeletal disorders have been published [24].

##### Outcome variables

A diagnosis of hip osteoarthritis was identified by the presence of ICD-9 diagnostic codes rendered by a medical provided and documented in the patient’s medical records within 24 months after surgery, and included 715.15, 715.25, 715.35, and 715.95. These codes were valid through June 2015, within the timeframe that this data was collected. This methodology has been utilized to identify incidence rates of osteoarthritis in this same population [26, 27].

##### Predictor variables

For the final model we selected variables a priori that were known to have a relationship with osteoarthritis, to include age (risk is expected to increase with age), sex (higher incidence reported in females), [28] and active duty military status (higher incidence reported in active duty military) [29]. We also a priori identified two variables present after surgery with clinical rationale for a potential relationship with osteoarthritis: surgical complications and exposure to additional hip surgeries (e.g. revision, arthroplasty, etc.).

#### Statistical approach

We first reported the prevalence of an OA diagnosis found in this cohort within 2 years of surgery. Descriptive characteristics, including means, standard deviations and frequencies were calculated for 2 groups: 1) those with a diagnosis of OA and 2) those without. Descriptive data included patient level demographics, pre and postoperative variables (e.g. use of opioids or physical rehabilitation, medical comorbidities), and surgical variables (e.g. revisions, complications).

Given the nature of the dependent variable (OA diagnosis within 2 years), a binary logistic regression was performed to identify relevant predictor variables [30]. A logical clustering of two sets of variables based on clinical significance was employed using both a sequential and hierarchical approach. We chose variables with logical clinical significance. There were very small counts for individual complications, so they were bundled together, with 2.9% (*n* = 55) having 1 or more complications, and 12.2% (*n* = 228) undergoing an additional hip surgery. The measure of association between these two variables was relatively low (phi = .071), indicating poor likelihood of multicollinearity. For those who had complications, 74.5% (*n* = 41) did not have additional surgery, hence both variables were included in the predictive model [31].

The first step included variables leading up to the surgical event (age, sex, and active duty military status) and the second step included variables occurring after the index surgery (complications and additional hip surgeries). This afforded the opportunity to assess to what extent the addition of post-surgical variables (when controlling for the set of pre-operative predictors) improved model fit. All parameter and pseudo r^2^ estimates were reported. The Hosmer-Lemeshow was used to assess model goodness of fit, [32] with non-significance preferred. All assumptions (distribution of residuals, leverage, influence, etc.) were closely examined. Adjusted Odds Ratios with 95% confidence intervals were reported with a level of α = 0.05 set as significant. SPSS v24.0 (IBM Corp. Armonk, NY, USA) was used for all analyses.

## Results

**Fig. 1:**
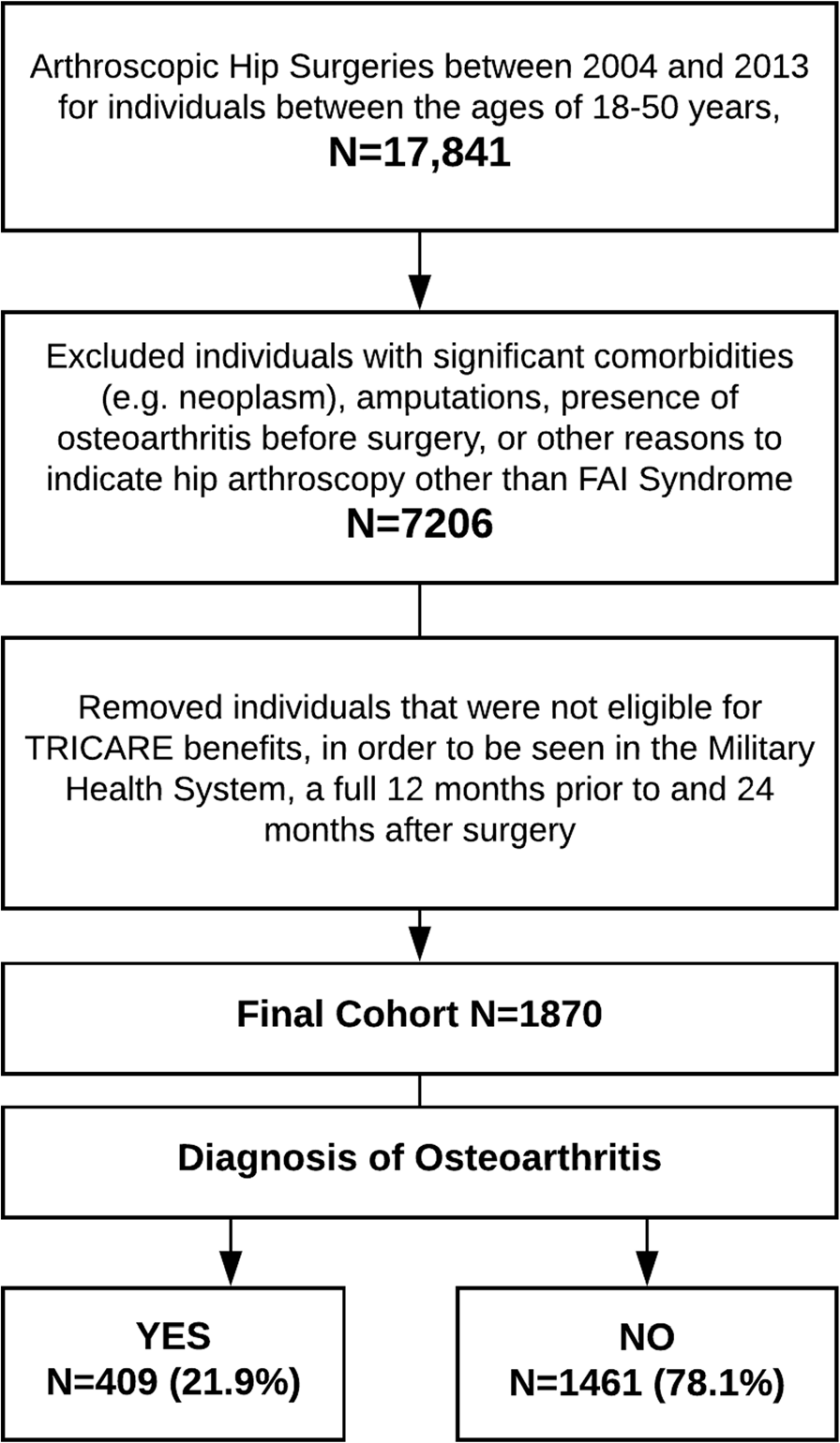
Identification of cohort study flow

There were 1870 participants included in the final cohort (Fig. 1), with a mean age of 32.24 years (SD = 8.09, range from 18 to 50) and 55.5% (*n* = 1037) were males (Table 1). Within 2 years of undergoing hip arthroscopy, 409 individuals (21.9%) had received a new clinical diagnosis of hip OA. Univariate comparison of clinical care variables in those with and without hip OA is reported in Table 2.

**Table 1:** Demographics of Cohort at Baseline Comparing With and Without Hip Osteoarthritis

	Hip OA Diagnosis YES 409 (21.9)	Hip OA Diagnosis NO 1461 (78.1)	*P*-value
Female Sex	151 (36.9)	682 (46.7)	< 0.001*
*Age Group – Mean (SD)*	34.1 (7.9)	31.7 (8.1)	< 0.001*
18–30 – N (%)	143 (35.0)	702 (48.0)	
31–40 – N (%)	167 (40.8)	507 (34.7)	
41–50 – N (%)	99 (24.2)	252 (17.2)	
*Beneficiary Category*			0.907
Active Duty	270 (66.0)	993 (68.0)	
Guard-Reserve	1 (0.2)	5 (0.3)	
Retired Service Member	5 (1.2)	17 (1.2)	
Dependent	94 (23.0)	326 (22.3)	
Other-unknown	39 (9.5)	120 (8.2)	
*Military Service (status of dependents based on service member)*			0.786
Army	176 (43.0)	657 (45.0)	
Air Force	102 (24.9)	365 (25.0)	
Navy	76 (18.6)	229 (15.7)	
Marine Corps	49 (12.0)	176 (12.0)	
Coast Guard	5 (1.2)	25 (1.7)	
Other	1 (0.2)	7 (0.5)	
Missing	0	2 (0.1)	
*Socioeconomic Status (status of dependents based on service member)*			0.028*
Junior Enlisted	84 (20.5)	388 (26.6)	
Senior Enlisted	226 (55.3)	717 (49.1)	
Junior Officer	42 (10.3)	190 (13.0)	
Senior Officer	53 (13.0)	152 (10.4)	
Unknown	4 (1.0)	14 (1.0)	
*Location of Surgery*			0.002*
Military Hospital	184 (45.0)	783 (53.6)	
Civilian Hospital	225 (55.0)	678 (46.4)	

Note: *P < 0.05; OA = osteoarthritis; N (%) unless otherwise noted

**Table 2:** Univariate Comparison of Clinical Care Variables in those With and Without Hip Osteoarthritis

	Hip OA Diagnosis YES N (%) - 409 (21.9)	Hip OA Diagnosis NO N (%) - 1461 (78.1)	*P*-value
Preoperative variables (PRESENT within 12 months prior to surgery) *N* (%)			
Hip-related Rehabilitation (at least 3 visits)	191 (46.7)	659 (45.1)	0.575
Opioid Use	180 (44.0)	581 (39.8)	0.125
Comorbidities			
Insomnia	35 (8.6)	130 (8.9)	0.845
Systemic Arthropathy	5 (1.2)	29 (2.0)	0.404
Substance Abuse	61 (14.9)	227 (15.5)	0.816
Mental Health	98 (24.0)	274 (18.8)	0.021*
Operative variables			
Surgical Procedures of Interest – *N* (%)			
Femoroplasty	112 (27.4)	348 (23.8)	0.153
Acetabuloplasty	75 (18.3)	241 (16.5)	0.412
Labral Repair	98 (24.0)	395 (27.0)	0.228
Postoperative variables (present within 24 months after surgery) *N* (%)			
Hip-related Rehabilitation (at least 3 visits)	353 (86.3)	1178 (80.6)	0.009*
Prescription Opioid Use	320 (78.2)	1019 (69.7)	0.001*
Comorbidities			
Insomnia	35 (8.6)	116 (7.9)	0.758
Systemic Arthropathy	20 (4.9)	59 (4.0)	0.486
Substance Abuse	111 (27.1)	342 (23.4)	0.133
Mental Health	185 (45.2)	515 (35.2)	< 0.001*
Complications			
Infection	2 (0.5)	3 (0.2)	0.301
Avascular Necrosis	5 (1.2)	3 (0.2)	0.015*
Fracture	8 (2.0)	25 (1.7)	0.832
Heterotopic Ossification	2 (0.5)	10 (0.7)	1.000
Additional Hip Surgery (e.g., revision)	81 (19.8)	147 (10.1)	< 0.001*

Note: *P < 0.05, *OA* Osteoarthritis

At the first step of the model (pre-surgery variables), the overall model was significant:, *p* < 0.001; 0% Sensitivity (Sn), 100% specificity (Sp), 78.1% accuracy. Age (higher) and sex (male) were significant predictors for an OA diagnosis (Table 3). At the second step of the model, adding the post-surgical variables improved the model (*p* < 0.001) and the overall model fit (i.e., all five predictors) was also significant (p < 0.001). With the 5-predictor model (Table 3), there was 0.2% (Sn) and 99.7% (Sp) with an overall accuracy of 77.9%. The following individual variables were significant: (1) age (OR = 1.04; 95% CI = 1.02, 1.05), (2) male sex (OR = 1.31; 95% CI = 1.04, 1.65), and (3) having undergone an additional hip surgery (OR = 2.33; 95% CI = 1.72, 3.16). Neither active duty military status nor complications were significant predictors of a new clinical diagnosis of OA (Table 3).

**Table 3:** Adjusted Odds Ratios for Variables in Model Predicting Hip Osteoarthritis Diagnosis 24 months After Surgery

	Odds Ratio	95% CI	*P*-Value
Step 1: Variables present at baseline Hosmer & Lemeshow *p* = 0.445; Cox R^2^ = 0.018; Nagelkerke R^2^ = 0.028			
V1: Age	1.037	1.023 to 1.052	< .001*
V2: Military Status (Active Duty)	1.117	0.881 to 1.416	0.361
V3: Sex (male)	1.305	1.035 to 1.645	0.025*
Step 2: Variables present after surgery Hosmer & Lemeshow *p* = 0.826; Cox R^2^ = 0.033; Nagelkerke R^2^ = 0.051			
V4: Surgical Complications	1.136	0.613 to 2.104	0.686
V5: Underwent an Additional Hip Surgery	2.330	1.718 to 3.159	< .001*

There was a significant effect when adding Step 2 variables to the model after Step 1 (χ^2^(2) = 28.48, *p* < .001). The parameter estimates are from the 2nd step of variable entry (all 5 variables). *V* Variable, *CI* Confidence Interval; *P < 0.05

## Discussion

The primary aim of this study was to identify the proportion of individuals that received a new clinical diagnosis of hip OA within 2 years of arthroscopic hip surgery, which in this cohort was 21.9%. These findings suggest that a clinical diagnosis of OA can occur within as little as 2 years after arthroscopic hip-preservation surgery in younger adults without a pre-surgery diagnosis of OA. We also identified variables that predicted a clinical diagnosis of OA after surgery, which included age, sex, and having undergone an additional surgery. However, the predictors were not very strong, and only 1 of the 3 is potentially modifiable (additional surgeries). Caution is warranted with using these variables to predict OA after surgery. While associations between comorbidities and OA have been identified, for example mental health [33, 34] or diabetes, [35] there is no evidence to support their ability to predict onset of OA diagnosis and therefore these were not included in our model. Finally, other factors that were not captured within the data source utilized for this study may be of value and have stronger predictive capability (e.g. past history of injury, response to conservative treatment, radiographic baseline, characteristics of joint morphology, and specific prior physical demands based on military specialty or occupation).

The rate of 21.9% with a clinical diagnosis of OA is lower than reports of radiographic hip OA found within 24 months after hip arthroscopy (37%), but in a slightly younger (mean age of 32 vs 37 years) and much larger cohort (*N* = 1870 vs 70) [6]. This may not be any different than the expected rate of radiographic OA findings in individuals with asymptomatic FAI, [15] albeit in older cohorts f (mean age 49.3 years). The incidence of OA among military service members in general, and especially of the hip, appears to be higher than the non-military population [27, 36]. High-level occupation-related physical demands, common in the military, and especially activities involving frequent and repetitive bending, squatting, kneeling, lifting, and climbing, have all been associated with hip OA [37, 38]. It is unknown if surgical intervention can influence these rates (i.e. reduce progression). However, our cohort included both active duty and civilian dependents, and military status was not a significant predictor of a clinical diagnosis of OA in the 2 years following hip arthroscopy.

Our findings suggest that females were at lower risk, while older age brought on greater risk for receiving a clinical diagnosis of hip OA after hip arthroscopy. Disease characteristics of FAI syndrome are reported to vary between males and females, [39] and this has the potential to influence onset and progression of OA. Another explanation for the lower rate in females is that greater loading of the hips increases the risk for developing OA, [40, 41] and during the period of surveillance in this study, females were not authorized in combat roles and therefore in positions with less load carriage requirements. In the 5th and 6th decade, rates of hip OA are higher for women, but before then, they appear to be higher in men [42]. Specific to the relationship between hip OA and FAI syndrome, previous cohorts have had older participants (mean ages of 55.9 years, [4, 5] 62 years, [43] 54 years, [44] 70.6 years, [45] 37 years for entire cohort - age of individuals with OA not reported, [6] and median age of 54 years [46]). The mean age in our cohort was 32 years, and less is known about younger patients. Further research is needed to clarify this relationship, especially in younger individuals.

The method for identifying osteoarthritis is also important to consider. Large epidemiological studies of OA, such as the estimates of global burden of hip and knee osteoarthritis, [28] report utilizing 4 methods for the identification of osteoarthritis: 1) radiographic OA with symptoms, 2) radiographic OA regardless of symptoms, 3) self-report of OA, and 4) clinical diagnosis rendered by a medical professional. Estimates and comparisons are challenging, due to known disparities between these different methodologies. The majority of studies assessing the relationship between OA and FAI syndrome to date have used radiographic criteria [4–6, 43, 45–47]. We utilized a clinical diagnosis rendered by a medical provider. Given that only ~ 25% of individuals with radiographic OA also have clinical symptoms, [28] it is likely that rates of OA would have been even higher in our cohort if radiographic criteria were utilized. Given all these findings, our methodology was more likely to have under rather than over-estimated the true rate of hip OA.

Having an additional surgery was also a factor that predicted a clinical diagnosis of OA in our model. Although no causality in these relationships can be implied, the fact that these individuals had an additional surgery suggests that the initial procedure was not satisfactory, and/or the extent of the pathology was more extensive. Additional surgeries also provided more interactions with the health system, and potentially more opportunities for an OA diagnosis to be rendered. Individuals undergoing additional surgeries also utilized more opioids and more physical rehabilitation, which may explain the higher utilization rates of each (opioids and physical therapy) in those with a diagnosis of OA.

Hip arthroscopy is a technically demanding procedure, [48, 49] with documented inconsistency in description of surgical indications, surgical technique and subject demographics [50, 51]. The extent to which the variability of this treatment contributes to the difference in a OA diagnosis is unclear. If hip arthroscopy does indeed slow down progression of hip OA, then the experience of the surgeon or the procedure type could also potentially influence outcomes. For example, cam deformities are more strongly correlated to hip OA than pincer deformities, significantly predicting hip OA within 5 years. [47]. These variables were not available with this data set. In 2011 new CPT procedure codes, one each for cam and pincer lesions, as well as labral repair procedures codes became available, but system-wide adoption was likely not immediate and would have affected only the tail end of our cohort. The availability of this data for inclusion in our model would have been very insightful. The counter-argument is that surgical correction itself may be associated with structural progression of hip OA [18]. Arthroscopy can accelerate the progression of arthritis [52]. Hemarthrosis and even a single episode of intra-articular bleeding can lead to joint damage, adversely affect cartilage health, and potentially recapitulate and prolong the events initiated by the primary trauma [53, 54]. Findings in asymptomatic individuals, including athletes with morphological changes and labral tears [20], along with reports that as many as 80% of non-surgical hips with morphology attributed to FAI Syndrome had not developed OA over a mean of 18.5 years after initial assessment, [15] suggest that some individuals will not develop hip OA, despite absence of surgical intervention. The lack of long-term, high quality studies supporting hip preservation surgery as a valid preventative procedure for hip OA are a necessary discussion point with patients if prevention of OA is one of the goals. Finally, improved methods for identifying early OA and measuring progression of the disease continue as significant gaps that prevent a clear understanding of the association between hip morphology and hip osteoarthritis onset.

These findings do not imply that OA is a caused by arthroscopic surgery, but rather that it is not uncommon to find a diagnosis rendered within as little as two years after surgery. The 21.9% incidence of an osteoarthritis diagnosis after surgery in this cohort after only 2 years is similar to rates of OA occurrence in patients with FAI Syndrome treated non-surgically over a mean of 18.5 years [15]. If our cohort were followed for 18.5 years, the incidence would likely be much higher. Prospective matched controls of younger individuals with and without FAI, and with and without surgery are needed to appropriately compare incidence rates and adequately determine whether the hip joint can be better-preserved over time through surgical intervention.

### Limitations

There are several limitations. First of all, an OA diagnosis is based on a code in the patient medical record provided by a clinician. It is difficult to confirm what criteria was followed by the clinician to make the diagnosis. It likely included a combination of clinical and radiographic variables. Limited research exists regarding reliability and validity for case ascertainment using ICD diagnosis codes for osteoarthritis, and their value likely varies greatly depending on setting and type of diagnosis. For example, ICD codes are valid (95% sensitivity; 96% specificity) for identifying patients with knee or hip replacement in the Veterans Health Administration system [55]. Although there can be false-positives, they tend to underestimate the rate in other conditions such as obesity (15.1% of true obesity rates were identified) [56] or psoriasis (81% valid case ascertainment) [57]. The sensitivity for identifying 32 different conditions using ICD-9 codes varied from 9.3 to 83.1% [58]. There is also no way to determine the severity of OA or its correlation with function/disability. Codes from medical records data have been used for surveillance of incidence rates in other studies within this population, [26, 27] although this methodology tends to underestimate the incidence rates of disease in general. This means that true rates of OA could be higher than 22%. Patients in our cohort were also more likely to have symptomatic OA, as our definition required that patients sought out medical care and then received a diagnosis from a medical provider. While no clinical diagnosis of OA was rendered in the year prior to surgery, we cannot determine with certainty that OA did not exist prior to surgery. It is possible and likely that some of these patients had undetected or undiagnosed osteoarthritis prior to surgery. The specific genesis of OA is still an area of developing investigation, with a general lack of early indicators that are clinically relevant. Despite our best attempts to limit inclusion to only cases of arthroscopy specific to FAI syndrome, it is possible some of the arthroscopic cases were for other reasons. Occupational requirements vary across many various military positions, and therefore some service members could be more susceptible than others to higher and more repetitive loading of the hip. Unfortunately, we could not determine military occupational specialty from within MDR. However, this may have played less of a role, as there was no difference in clinical OA diagnosis rendered between military service members and civilians within our cohort. Finally, our cohort only included those that were still eligible TRICARE beneficiaries 2 years after surgery. Three times as many individuals that had surgery were not included in the cohort because they were not available for a minimum of 2 years after surgery (e.g. medical separation, retirement, end of enlistment, etc.). It is possible incidence rates could be different if all of these individuals were included. For those who were included, their healthcare utilization was only captured out to 2 years. Therefore, only diagnoses of OA within 2 years are represented. It is likely many more developed OA after 2 years. One study reported that hip OA could take more than 10 years to manifest [46].

## Conclusion

A little over 20% of younger adults with no prior diagnosis of osteoarthritis that underwent hip arthroscopy for FAI syndrome received a clinical diagnosis of OA within 2 years of surgery. These rates may differ when using alternate criteria to define OA, such as radiographs. Estimating OA disease progression after hip arthroscopy for FAI syndrome in young adults is important and should take into consideration the various surveillance options. Each one has its downside, but a more comprehensive approach will likely be necessary to accurately identify the true rates of prevention of joint disease onset or disease progression that arthroscopic surgery is claimed to provide. Prospective trials with sound methodology are needed to determine accurate rates and predictors of osteoarthritis onset after hip preservation surgery.

## Data Availability

Data from the Military Health System Data Repository (MDR) s proprietary to the US Defense Health Agency and can be made available after obtaining an approved Data Sharing Agreement from the DHA Privacy Board (further information can be found on health.mil).

## Abbreviations

CPT: Current Procedural Terminology
DHA: Defense Health Agency
DoD: Department of Defense
FAI: Femoroacetabular Impingement
IBM: International Business Machines
ICD: International Classification of Diseases
MDR: Military Health System Data Repository
MHS: Military Health System
OA: Osteoarthritis
RECORD: REporting of studies Conducted using Observational Routinely collected health-Data
Sn: Sensitivity
Sp: Specificity
SPSS: Statistical Package for Social Sciences
STROBE: Strengthening of Reporting of Observational Studies in Epidemiology
